# Research on Fingerprint Map Construction and Real-Time Update Method Based on Indoor Landmark Points

**DOI:** 10.3390/s25175473

**Published:** 2025-09-03

**Authors:** Yaning Zhu, Yihua Cheng

**Affiliations:** 1 School of Mechanical Science & Engineering, Huazhong University of Science and Technology, Wuhan 430074, China; 2 School of Computer Science, University of Birmingham, Birmingham B15 2TT, UK

**Keywords:** fingerprint map, location-based services, indoor positioning systems, INS

## Abstract

WIFI base stations have full indoor coverage, and the inertial navigation system (INS) is independent and autonomous, with high short-term positioning accuracy. However, errors accumulate over time, and an INS/WIFI combination has become the mainstream research direction regarding indoor positioning technology. The accuracy of WIFI fingerprint maps deteriorates significantly with changes in the environment or time, and there is an urgent need to solve the problem of automatic real-time updating of fingerprint maps. This article addresses the issue that the existing real-time acquisition technology for fingerprint point locations has severely restricted the real-time updating of fingerprint maps. For the first time, landmark points are introduced into the fingerprint map, and landmark point fingerprints are defined to construct a new fingerprint map database structure. A method for automatic recognition of landmark points (turning points) based on inertial technology is proposed, which achieves automatic and accurate collection of landmark point fingerprints and improves the reliability of crowdsourcing data. Real-time automatic monitoring of fingerprint signal fluctuations at landmark points and construction of error models have achieved real-time and accurate updates of fingerprint maps. Real scene experiments have shown that the proposed solution significantly improves the long-term stability and reliability of fingerprint maps.

## 1. Introduction

With the popularity of the Internet of Things and Internet of Everything, location-based services (LBSs) have become the basis of all social operations. Whether these are carried out by smart terminals, industrial sensors, or moving objects, they all rely on location information to achieve dynamic interaction and scene adaptation. GNSS does not have a line-of-sight (LOS) channel with indoor targets and so cannot meet indoor positioning needs. Additionally, people spend 80% to 90% of their time indoors [1,2]. In view of this, people are committed to researching and developing indoor positioning technology to solve the ‘last mile’ accurate positioning problem in Indoor Positioning Systems (IPSs) [2,3,4,5].

Indoor positioning can be divided into three categories: the first type is based on base station positioning, which achieves positioning through information exchange between devices and base stations. Wireless Fidelity (WiFi) [6,7] is where positioning is achieved by measuring parameters such as the Received Signal Strength Indication (RSSI) and propagation time for wireless Access Points (APs), which are susceptible to environmental interference. Bluetooth [8,9] is where positioning is achieved by measuring the strength of beacon broadcast signals, which are susceptible to electromagnetic interference or object obstruction. Ultra-Wide Band (UWB) [10,11] uses nanosecond pulse signals to achieve centimeter-level or even millimeter-level high-precision positioning; it has a strong anti-interference ability and is suitable for use in industrial-level high-precision positioning scenarios. Pseudo-Satellite (PL) [12,13]: Here, mobile devices receive simulated satellite signals to achieve positioning. The second type is based on perceptual positioning, where devices achieve positioning based on autonomously perceived information. Inertial Navigation System (INS) [14,15] uses an accelerometer to measure the linear acceleration and a gyroscope to measure the angular velocity, and the target position is calculated. Radar Location [16,17]: This is a wireless positioning method based on electromagnetic wave detection. Visual Positioning [18,19] collects image information using visual sensors, and localization is achieved using methods such as image processing and feature extraction. The third type [20,21,22,23,24,25] is combination positioning. WiFi positioning and the INS have their own advantages and disadvantages. In order to meet positioning needs in complex indoor environments, they are used together so that their advantages complement each other.

WIFI base stations can use existing network devices and provide full indoor coverage but require continuous updating of fingerprint maps [26,27]. The INS is independent, autonomous, and has high short-term positioning accuracy, but errors accumulate over time [28,29]. Intelligent mobile terminals integrate inertial sensors and are widely used [30]. Due to these factors, as well as people’s demand for low-cost and universal indoor positioning technology, an INS/WIFI combination has become the mainstream research direction regarding indoor positioning technology [31,32,33]. The signal strength sequence of an AP at the indoor fingerprint point has a unique quality, that is, a WIFI fingerprint signal. The WiFi fingerprint map is used to assist with the implementation of the INS and eliminate its accumulated error in real time.

The core of WIFI positioning is the construction and real-time updating of WIFI fingerprint maps. Due to changes in the device power, environment, and other factors, fingerprint signals change over time, and the current status of fingerprint maps must be maintained [34]. At present, there has been a lot of research on real-time update technology for fingerprint maps [34,35,36,37,38,39], but there are still problems. Firstly, the positional accuracy of the location labels attached to crowdsourced measurements is highly uncertain, leading to inaccurate models [40,41,42]. Secondly, in order to improve the positional accuracy of location tags, various restrictions are imposed, such as the need to use specialized devices [43,44], perform specific activities [45,46], and generate fingerprint maps that are difficult to process [47,48,49]. Finally, the GPR method [50,51] based on the use of crowdsourced fingerprint data has very limited scalability due to the limited positional accuracy of the fingerprint points.

A fingerprint map contains two core types of data; the first type is the WIFI fingerprint signal. The other type is the position coordinates of the fingerprint signal point. From the previous analysis, it can be seen that the difficulty in developing real-time update technology for WIFI fingerprint maps is the accurate acquisition of fingerprint point location information. In view of this, this article proposes a fingerprint map construction and real-time update method based on indoor landmark points. The main contributions are summarized as follows:

(1) Landmark points are used for the first time in fingerprint mapping.

Landmark point features: Their location remains unchanged. They possess unique characteristics that can be recognized by sensors, such as turning points, staircases, elevators, entrance and exit signs, etc. The fingerprint signals collected at landmark points are referred to as landmark point fingerprints.

(2) An automatic landmark point recognition method is developed.

An automatic turning point (landmark point) recognition method based on inertia technology is proposed, which achieves automatic and accurate acquisition of WIFI fingerprints (landmark point fingerprints) to improve the reliability of crowdsourced data.

(3) A novel fingerprint map database structure is developed.

The new structure incorporates an additional layer on top of the existing fingerprint map structure, which is used to store landmark point fingerprint maps.

(4) A real-time and relatively accurate fingerprint map updates to achieve reliable indoor positioning.

Based on regional landmark point fingerprints, real-time automatic monitoring of landmark point fingerprint signal fluctuations is carried out and an error model is constructed. When the fluctuation of local landmark point fingerprint signals reaches a certain threshold, all fingerprint signals within the region are automatically updated based on the error model to improve the long-term stability and reliability of the fingerprint map.

The remainder of this article is organized as follows. The Section 2 reveals the inconsistent positioning accuracy of traditional static fingerprint maps in long-term deployment through the analysis of annual measured data. Then, in the Section 3, we propose a novel fingerprint map structure based on indoor landmark points. The Section 4 presents a real-time update method for fingerprint maps based on landmark fingerprint signals. Section 5 shows the experiments and analysis, and Section 6 draws the conclusions.

## 2. Time Varying Experiment of Fingerprint Maps

In free space, the propagation model of WIFI signal strength is as follows:

(1) $$rss_{i}^{j}=P_{i}^{j}-10\alpha_{i}^{j}\mathrm{lg}d_{i}^{j}+X_{i}^{j}$$

where *i* is the fingerprint point number (*i* = 1, 2, …, n); *j* is the AP number (*j* = 1, 2, …, m); *α* is the path loss coefficient; *P* is the emission power; *d* is the distance of the propagation path; and *X* represents a zero-mean Gaussian random variable, which is the average received power change caused by environmental changes. From this model, it can be seen that the fluctuation in WIFI signal strength is influenced by natural environmental factors, spatial environmental factors, and the factors of the WIFI signal transmission source itself.

This section explores the time-varying characteristics of fingerprint maps through experiments and analyzes the impact of the time-varying nature of WIFI fingerprint maps on positioning accuracy.

### 2.1. WIFI Fingerprint Signal Fluctuation Experiment

**Figure 1:**
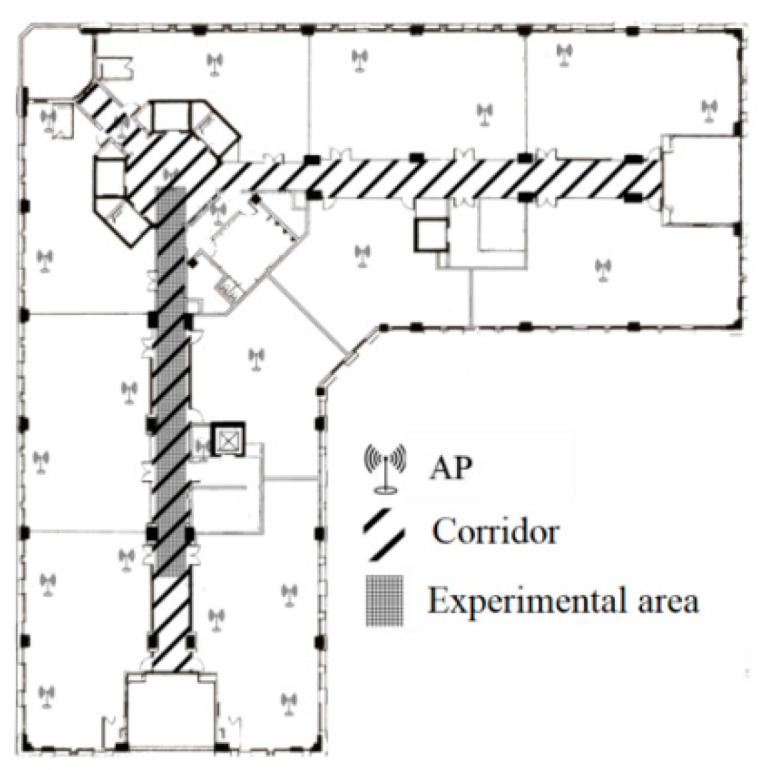
Experimental area plan (size, long—55 m, wide—55.6 m).

The experimental area is set as a corridor of a building (Figure 1), with a width of about 2 m and a length of about 10 m. There are 22 fingerprint points evenly distributed in the experimental area, with a spacing of 1 m between them. The experiment lasted from 1 January to 31 December 2023, during which all APs worked normally, with their positions and antenna orientations unchanged. The experimental period was one year, and the measured fingerprint signals from 12 February, 10 April, 15 August, and 13 December were selected for analysis.

**Figure 2:**
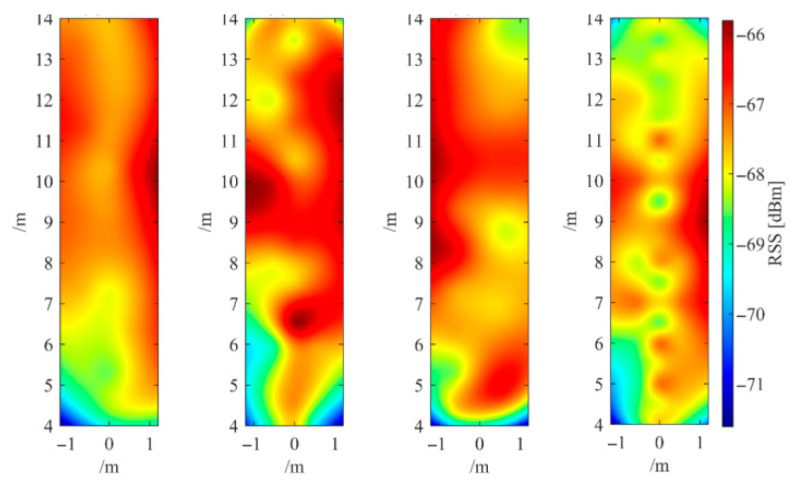
Distribution of fingerprint overlay signal strength within the experimental area.

To verify the time-varying characteristics of fingerprint signals, first, the superimposed signal strength *RSS_i_* of fingerprint points is taken as the observed quantity, as shown in Formula (2). An interpolation method is used to convert the superimposed signal strength of discrete fingerprint points into a continuous regional distribution map, as shown in Figure 2, from left to right, which are the measured data on 12 February, 10 April, 15 August, and 13 December, respectively. From the four images in Figure 2, it is evident that the fingerprint signal exhibits significant temporal variability.

(2) $$\sum RSS_{i}=rss_{i}^{1}+rss_{i}^{2}+\ldots +rss_{i}^{j}+\ldots +rss_{i}^{m}$$

Then, the time-varying fingerprint signals of each test point were analyzed, and AP signal values from 1 to 40 were collected at the test points, as shown in Table 1, Table 2, Table 3 and Table 4.

**Figure 3:**
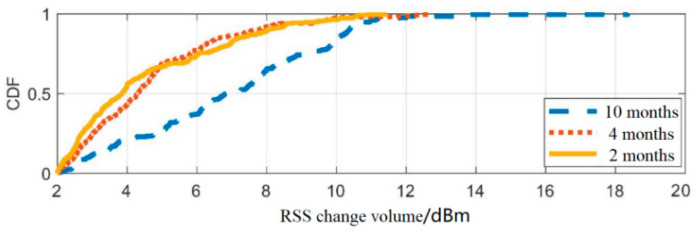
AP signal CDF distribution map.

Finally, the Cumulative Distributed Function (CDF) is applied to further analyze the time-varying characteristics of the fingerprint signal, as shown in Figure 3, and the data statistics table is presented in Table 5.

From Figure 3 and Table 5, it can be seen that when the data collection interval is 2 or 4 months, the CDF curves of RSS are similar, and the statistical measures are similar. When the time interval is 10 months, the CDF curve undergoes significant changes, and the statistical mean is doubled, indicating that the timeliness of fingerprint signals significantly affects the accuracy of fingerprint maps.

### 2.2. The Impact of WiFi Fingerprint Signal Fluctuation on Positioning Accuracy

**Figure 4:**
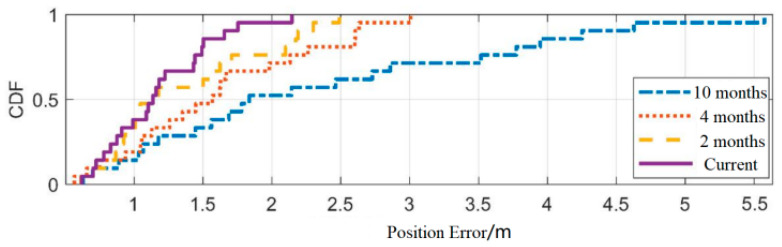
Fingerprint map positioning error CDF.

The experimental area is shown in Figure 1, and the fingerprint localization method is derived from reference [52]. Perform positioning accuracy analysis on static fingerprint maps constructed on 12 February, 10 April, 15 August, and 13 December using the same set of test data. The test results are shown in Figure 4.

As shown in Figure 4, based on the latest fingerprint map constructed on that day, the positioning accuracy error CDF is distributed within 1.65 m. The update time of the fingerprint map is two months apart, and the positioning accuracy error CDF is distributed within 2 m. With four months apart, CDF is within 2.5 m, and with ten months apart, CDF is within 5.2 m. From the last column of Table 6, it can be seen that using fingerprint maps with an interval of 10 months, 20% of the test data had positioning errors exceeding 4.26 m.

Based on the above analysis, the WIFI fingerprint map needs to continuously optimize signal coverage and update the fingerprint database.

## 3. New Fingerprint Map Based on Indoor Landmark Points

Bahl et al. [52] correlated WIFI signals with fingerprint point coordinates and constructed a fingerprint map for the first time. This article addresses the challenge of accurately obtaining fingerprint point location information in real-time updates of WIFI fingerprint maps. For the first time, the landmark point fingerprints are proposed, which adds the landmark point fingerprint layer to the existing fingerprint map architecture and constructs a new type of fingerprint map. Below is an introduction to the new fingerprint map architecture.

### 3.1. Traditional Fingerprint Map Layer

Each WIFI fingerprint signal is shown in Equation (3), which consists of the coordinates of the fingerprint point and the strength of the AP signal at the fingerprint point.

(3) $$L-rss_{i}=[(x_{i},y_{i}),(rss_{i}^{1},rss_{i}^{2},\ldots ,rss_{i}^{j},\ldots ,rss_{i}^{n})]$$

where, *i* is the fingerprint signal of the *i*-th fingerprint point; *j* is the *j*-th AP; and $\left(x_{i},y_{i}\right)$ are the plane coordinates of the *i*-th fingerprint point.

The WIFI fingerprint signal layer R is composed of all WIFI fingerprint signals which are uniformly distributed indoors, as shown in Equation (4), where m is the number of fingerprints contained in the signal layer.

(4) $$R=\left[\begin{array}{c} L-rss_{1}\\ L-rss_{2}\\ \vdots \\ L-rss_{i}\\ \vdots \\ L-rss_{m} \end{array}\right]=\left[\begin{array}{cccccccc} x_{1} & y_{1} & rss_{1}^{1} & rss_{1}^{2} & \ldots & rss_{1}^{j} & \ldots & rss_{1}^{n}\\ x_{2} & y_{2} & rss_{2}^{1} & rss_{2}^{2} & \ldots & rss_{2}^{j} & \ldots & rss_{2}^{n}\\ \vdots & \vdots & \vdots & \vdots & \ldots & \vdots & \ldots & \vdots \\ x_{i} & y_{i} & rss_{i}^{1} & rss_{i}^{2} & \ldots & rss_{i}^{j} & \ldots & rss_{i}^{n}\\ \vdots & \vdots & \vdots & \vdots & \ldots & \vdots & \ldots & \vdots \\ x_{m} & y_{m} & rss_{m}^{1} & rss_{m}^{2} & \ldots & rss_{m}^{j} & \ldots & rss_{m}^{n} \end{array}\right]$$

### 3.2. Landmark Point Fingerprint Map Layer

**Figure 5:**
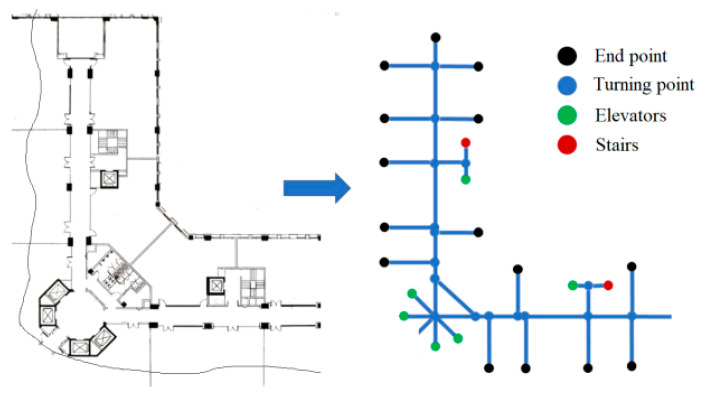
Experimental area and its abstract post link model.

According to the recognition ability of sensors, there may be different types of landmark points. For example, safety exits, doors, windows, etc., can serve as landmark points for visual sensors. Elevators, stairs, and turning points are types of inertial sensor landmark points. Based on the link model proposed by Alibaba Intelligent Multi-scenario Collaborative (ALIMC), the indoor area is abstracted. Figure 5 shows an experimental area and its abstracted link model.

According to the link model, the landmark points selected in this paper can be automatically recognized by inertial sensors. Table 7 provides the definition and attributes of landmark points. Type: 1 represents elevators or stairs, 2 represents turning points, and 3–6 are reserved attributes. Area: This attribute is used for quick retrieval of landmark points and real-time updating of fingerprint maps by region. Heading: this is a proprietary attribute of landmark turning points. All landmark point fingerprints form the landmark layer *H*.

Each WIFI landmark point fingerprint signal is shown in Equation (5).

(5) $$LL-rss_{i}=[(x_{i},y_{i}),(rss_{i}^{1},rss_{i}^{2},\ldots ,rss_{i}^{j},\ldots ,rss_{i}^{n}),(Type,Area,Heading)]$$

(6) $$H=\left[\begin{array}{c} LL-rss_{1}\\ LL-rss_{2}\\ \vdots \\ LL-rss_{i}\\ \vdots \\ LL-rss_{m} \end{array}\right]=\left[\begin{array}{cccccccc} x_{1} & y_{1} & rss_{1}^{1} & rss_{1}^{2} & \ldots & rss_{1}^{j} & \ldots & rss_{1}^{n}\begin{array}{cccc} & Type_{1} & Area_{1} & Heading_{1} \end{array}\\ x_{2} & y_{2} & rss_{2}^{1} & rss_{2}^{2} & \ldots & rss_{2}^{j} & \ldots & rss_{2}^{n}\begin{array}{cccc} & Type_{1} & Area_{1} & Heading_{1} \end{array}\\ \vdots & \vdots & \vdots & \vdots & \ldots & \vdots & \ldots & \vdots \\ x_{i} & y_{i} & rss_{i}^{1} & rss_{i}^{2} & \ldots & rss_{i}^{j} & \ldots & rss_{i}^{n}\begin{array}{cccc} & Type_{1} & Area_{1} & Heading_{1} \end{array}\\ \vdots & \vdots & \vdots & \vdots & \ldots & \vdots & \ldots & \vdots \\ x_{m} & y_{m} & rss_{m}^{1} & rss_{m}^{2} & \ldots & rss_{m}^{j} & \ldots & rss_{m}^{n}\begin{array}{cccc} & Type_{1} & Area_{1} & Heading_{1} \end{array} \end{array}\right]$$

**Figure 6:**
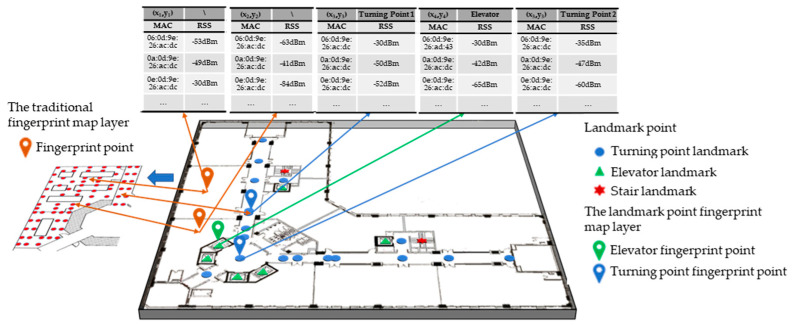
A new fingerprint map based on landmark points.

The landmark point layer (Equation (6)) and signal layer (Equation (4)) are associated by position coordinates to construct a new fingerprint map based on landmark points, as shown in Figure 6.

## 4. Real-Time Updating of Landmark Point Fingerprint Maps

Real-time updates based on landmark point fingerprint maps have two core modules: one is the landmark point recognition module. The other is the fingerprint map update module.

### 4.1. Recognition Module

**Figure 7:**
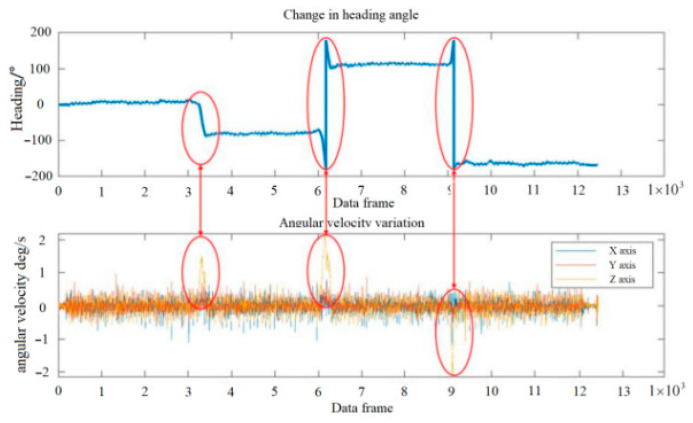
Inertial technology raw data and heading calculation results.

In indoor environments, the three types of landmarks, namely turning points, stairs, and elevators, are most widely distributed and have the highest frequency of user contact. It is relatively straightforward to detect signs such as elevators and stairs based on acceleration sensors. Here, we will focus on the method of identifying turning point landmarks based on inertial technology. When pedestrians carry smart mobile terminals through turning points, the angular velocity around the direction of gravity will undergo significant changes. This article is based on the strap down method to extract pedestrian heading information, avoiding the posture impact caused by device placement status (such as pocket, handheld). The extracted heading angle changes are shown in Figure 7.

**Figure 8:**
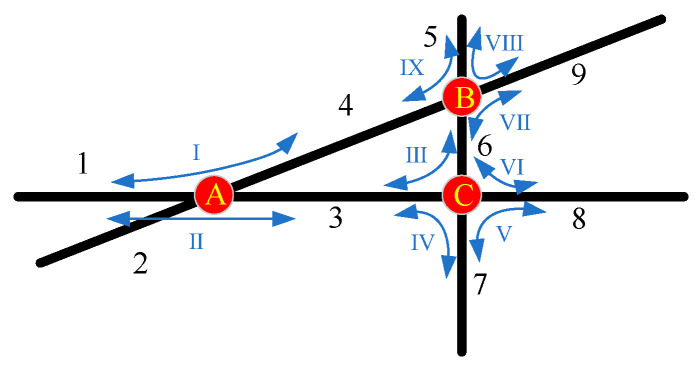
Diagram of crossing nodes.

As shown in Figure 8, based on the link node model, nodes and links are its core elements, with nodes as turning points or endpoints. It includes nine links (1, 2, …, 9) and three nodes (A, B, C). Defining the journey from link 1(4) through node A to link 4(1) as a crossing I. There are a total of nine crossings (I, II, …, IX) in the picture, and the turning point is identified during the crossing process, as shown in Formulas (7) and (8)

(7) $$\Delta \theta_{t}=\theta (t)-\theta (t-1)$$

where t is the time of data sampling; $\Delta \theta_{t}$ is the difference in heading angle between t sampling time and (t − 1) sampling time; and θ(t) is the heading angle at t time. In theory, when traveling straight on the link, the heading angle remains constant at $\Delta \theta_{t}=0$. To avoid false positives caused by heading sway during motion, an even number of adjacent heading angle changes are used here, and the generalized likelihood ratio test is used for threshold detection. The inflection point judgment conditions are as follows:

(8) $$\frac{\sum_{i=t-3}^{t}{\left(\Delta \theta_{i}\right)}^{2}}{\sum_{i=t-3}^{t}{\left(\Delta \theta_{i}-\frac{1}{t-t_{0}}\sum_{i=t-3}^{t}\Delta \theta_{i}\right)}^{2}}>\lambda$$

where $t_{0}$ is the starting time of the crossing process and λ is the threshold for identifying turning points.

**Figure 9:**
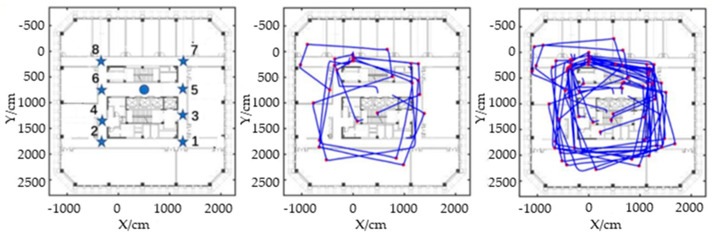
Experimental area and experimental trajectory diagram.

This article selects the indoor area shown in Figure 9 (right) to experimentally verify the method of identifying landmark points (turning points). This area contains eight turning points. The pedestrian movement trajectory of the first experiment is shown in Figure 9 (middle), during which the pedestrian passed through a total of 25 turning points. The pedestrian movement trajectory of the second experiment is shown in Figure 9 (left), during which the pedestrian passed through a total of 72 turning points.

From the statistical results in Table 8, it can be seen that in the first experiment, pedestrians passed through turning point 1 three times, turning point 2 three times, turning point 3 once, turning point 4 twice, turning point 5 three times, turning point 6 three times, turning point 7 five times, and turning point 8 five times. The automatic inflection point recognition method achieved 100% recognition of pedestrians passing through turning points. In the second experiment, pedestrians passed through turning point 1 11 times, turning point 2 11 times, turning point 3 times, turning point 4 twice, turning point 5 10 times, turning point 6 times, turning point 7 times, and turning point 8 15 times. The automatic inflection point recognition method achieved 100% recognition of pedestrians passing through turning points.

### 4.2. Update Module

**Figure 10:**
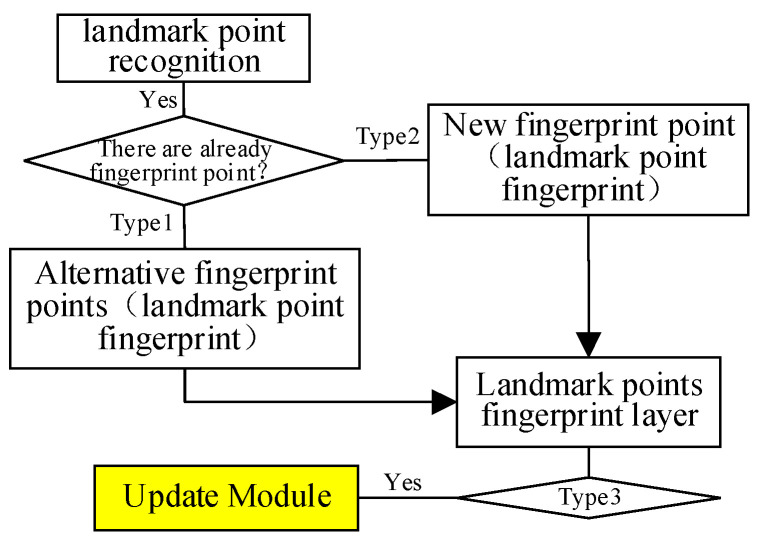
Real-time update process of fingerprint map.

**Figure 11:**
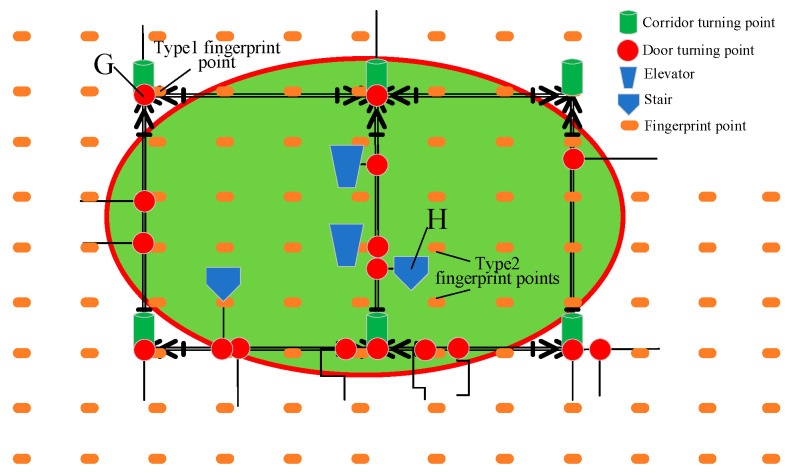
Distribution of landmark point fingerprints in the new fingerprint map.

The real-time update process of the fingerprint map is shown in Figure 10, which is divided into three categories. Type 1: When fingerprint points coincide or are close to landmark points, such as landmark point G in Figure 11, the newly collected landmark point fingerprint signal is directly used to replace the original fingerprint signal. Type 2: If the fingerprint point does not coincide or is not close to the landmark point, such as landmark point H in Figure 11, a new landmark point fingerprint will be added to the landmark point layer. Type 3: As shown in the elliptical area in Figure 10, the area contains rich landmark point fingerprints. The update module detects the fluctuation of landmark point fingerprint signals in real time. When the fingerprint signal fluctuation reaches a certain threshold, the update module will construct an error model based on the fluctuation of all landmark point fingerprint signals in the area, as shown in Formulas (9)–(12).

(9) $$rss_{i-new}=\left(rss_{i-new}^{1},rss_{i-new}^{2},\ldots ,rss_{i-new}^{j},\ldots ,rss_{i-new}^{n}\right)$$

(10) $$rss_{i-old}=\left(rss_{i-old}^{1},rss_{i-old}^{2},\ldots ,rss_{i-old}^{j},\ldots ,rss_{i-old}^{n}\right)$$

(11) $$\Delta rss_{i}=\left(\Delta rss_{i}^{1},\Delta rss_{i}^{2},\ldots ,\Delta rss_{i}^{j},\ldots ,\Delta rss_{i}^{n}\right)$$

where $rss_{i-new}$ is the latest fingerprint signal of the i-th landmark point in the elliptical region and $rss_{i-new}^{j}$ is the strength of the newly collected j-th AP signal. $rss_{i-old}$ represents the old fingerprint signal at the i-th landmark in the elliptical region, $rss_{i-old}^{j}$ represents the strength of the j-th AP signal collected, and i, j, and n are natural numbers.

The error model can be obtained from Formula (1):

(12) $$\begin{array}{ll} \Delta rss_{i}^{j}=rss_{i-new}^{j}-rss_{i-old}^{j} & =(P_{i-new}^{j}-10\alpha_{i-new}^{j}\mathrm{lg}d_{i-new}^{j}+X_{i-new}^{j})\\ & -(P_{i-old}^{j}-10\alpha_{i-old}^{j}\mathrm{lg}d_{i-old}^{j}+X_{i-old}^{j})\\ & =\Delta P_{i}^{j}-10\Delta \alpha_{i}^{j}\mathrm{lg}d_{i}^{j}+\Delta X_{i}^{j} \end{array}$$

where $\Delta \alpha_{i}^{j}$ is the change in path loss coefficient and $\Delta X_{i}^{j}$ is the change in average received power, which are mainly influenced by the environment. $\Delta P_{i}^{j}$ is the change in transmission power, which can be approximated as a linear change. The distance d between the AP point and the fingerprint point is a fixed value.

Using Formula (12), fit $\Delta \alpha_{i}^{j}$, $\Delta X_{i}^{j}$, and the change in transmission power $\Delta P_{i}^{j}$ using the least squares method, and update all fingerprint signals within the elliptical region.

## 5. Experiment and Results

### 5.1. Experiment Scenario and Setting

The mobile terminal uses the HUAWEI Pura 70 Pro smartphone, which is manufactured by Huawei in Shenzhen, China. And the experimental area is shown in Figure 1, including corridors and rooms. About 80 APs were distributed in the experimental area, and 50 APs with high signal strength and activity were selected during the experiment. The experiment was conducted at four time points, namely on 12 February (first group experiment), 10 April (second group experiment), 15 August (third group experiment), and 13 December (fourth group experiment).

Three types of fingerprint maps were chosen, the first being the Initial Static Fingerprint Map (ISFMap). To verify the effectiveness of the real-time update method for fingerprint maps proposed in this article, fingerprint points were uniformly distributed at intervals of 1 m × 1 m in the experimental area. On 12 February, fingerprint signals were collected manually at the fingerprint points, with a 5-s interval, for a total of 20 times. The average value was taken as the fingerprint signal of the fingerprint point, and a fingerprint map of the experimental area was created. The second method was to Automatically Update the Fingerprint Map (AUFMap). Automatic updating of the fingerprint map is based on the initial static fingerprint map, which is automatically completed by the fingerprint map automatic updating module one day before the experimental time point. The third type was the Current Static Fingerprint Map (CSFMap). The map production process is similar to the initial static fingerprint map production process, but its completion time is one day before the experimental time point.

### 5.2. Result

**Figure 12:**
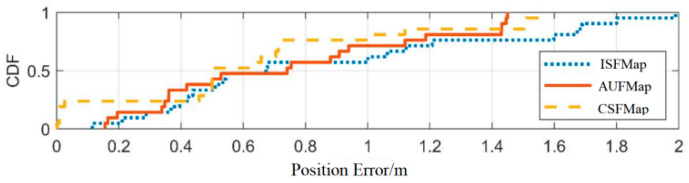
Distribution of positioning errors with a 2-month interval.

**Figure 13:**
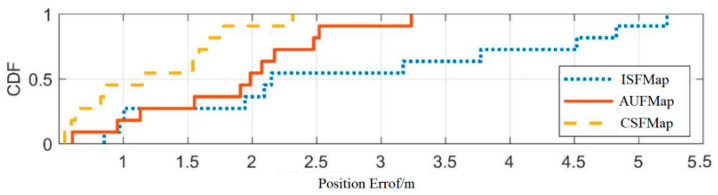
Distribution of positioning errors with a 6-month interval.

**Figure 14:**
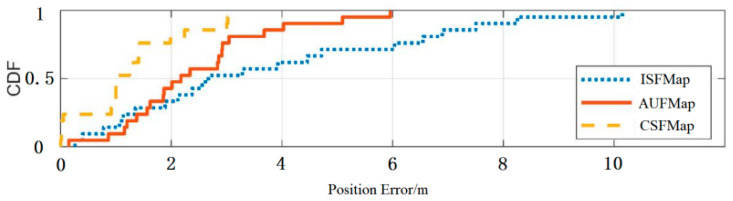
Distribution of positioning errors with a 10-month interval.

Based on four sets of experiments, the second set of experiments was compared with the first set of experiments (with an interval of 2 months). The third experiment was compared with the first experiment (with an interval of 6 months). The fourth experiment was compared with the first experiment (with an interval of 10 months). By comparing the error distribution curves and statistical values among the three, the experimental results were analyzed. The detailed comparison results are shown in Figure 12, Figure 13 and Figure 14 and Table 9, Table 10 and Table 11.

From Figure 12, Figure 13 and Figure 14, it can be seen that compared to ISFMap, using AUFMap significantly improves the positioning accuracy. The last column of Table 10 shows that 20% of the cases have positioning errors exceeding 3.67 m. Table 11 shows that 20% of the cases have positioning errors exceeding 7.32 m. The degradation of fingerprint map accuracy is mainly caused by the significant increase in positioning errors of some points, leading to the overall degradation of positioning accuracy. The new automatic update of fingerprint maps can effectively reduce these large error points, thereby ensuring the stability of the positioning system.

Meanwhile, it can be observed that the positioning accuracy of AUFMap is lower than that of CSFMap. This is because the update method updates the fingerprint signal based on the error model, which has lower accuracy compared to manual acquisition. However, compared to ISFMap, AUFMap still achieves a significant improvement in its accuracy. In addition, it can be found from Table 6, Table 7 and Table 8 that regardless of the time interval, the positioning accuracy of AUFMap has a certain degree of stability.

## 6. Conclusions

This article focuses on the problem of degradation of fingerprint map accuracy over time. Through the analysis of annual measured data, it reveals the inconsistent positioning accuracy of traditional static fingerprint maps in long-term deployment. A new fingerprint map structure based on indoor landmark points is proposed, which introduces landmark point fingerprints into the fingerprint map for the first time, overcoming the problem of strong uncertainty in the accuracy of fingerprint point positions in crowdsourced data. A real-time update method for fingerprint maps based on landmark point fingerprint signals has been proposed. Real scene experiments have shown that the proposed solution significantly improves the long-term stability and reliability of fingerprint maps.

## Figures and Tables

**Table 1:** Fingerprint data (12 February).

AP	MAC	Test Points			
		1 [dBm]	2 [dBm]		6 [dBm]
1	*:66:f1	NaN	−61.19	…	NaN
2	*:68:35	−56.98	NaN	…	−63.85
3	*:69:54	NaN	NaN	…	NaN
…	…	…	…	…	…
39	*:ac:62	NaN	−62.10	…	NaN
40	*:ac:63	−63.95	NaN	…	NaN

**Table 2:** Fingerprint data (10 April).

AP	MAC	Test Points			
		1 [dBm]	2 [dBm]		6 [dBm]
1	*:66:f1	−58.35	−54.79	…	−59.43
2	*:68:35	−57.88	NaN	…	NaN
3	*:69:54	NaN	−63.56	…	NaN
…	…	…	…	…	…
39	*:ac:62	−53.54	−50.02	…	−59.87
40	*:ac:63	−60.00	−56.29	…	−62.97

**Table 3:** Fingerprint data (15 August).

AP	MAC	Test Points			
		1 [dBm]	2 [dBm]		6 [dBm]
1	*:66:f1	−59.89	−57.54	…	NaN
2	*:68:35	−57.98	NaN	…	−60.01
3	*:69:54	NaN	−62.24	…	NaN
…	…	…	…	…	…
39	*:ac:62	−56.36	−50.60	…	NaN
40	*:ac:63	−53.65	−59.53	…	−61.70

**Table 4:** Fingerprint data (13 December).

AP	MAC	Test Points			
		1 [dBm]	2 [dBm]		6 [dBm]
1	*:66:f1	−57.00	−65.22	…	−62.56
2	*:68:35	NaN	NaN	…	NaN
3	*:69:54	NaN	NaN	…	NaN
…	…	…	…	…	…
39	*:ac:62	−58.66	NaN	…	NaN
40	*:ac:63	−55.34	−62.22	…	−56.40

Note: To simplify Table 1, Table 2, Table 3 and Table 4. * is 06:0d:9e:26. … is an ellipsis, the ellipsis on the 5th line omits the relevant data information for 35 AP points from the 4th to the 38th. The ellipsis in the fifth column omits the relevant data information for the third, fourth, and fifth test points.

**Table 5:** AP signal time-varying statistics.

Interval Time [Mon.]	Mean Value [dBm]	Median [dBm]	Standard Deviation [dBm]	80% Percentile [dBm]
2	2.35	1.69	2.11	3.85
4	2.82	2.47	2.48	5.36
10	4.95	5.01	3.03	8.27

**Table 6:** Statistics of positioning accuracy.

Interval Time [Mon.]	Mean Value [m]	Median [m]	Standard Deviation [m]	80% Percentile [m]
10	1.97	1.56	1.49	4.26
4	1.23	1.14	0.75	1.41
2	0.84	0.66	0.57	1.01
Current	0.63	0.50	0.49	0.71

**Table 7:** Landmark point fingerprint signal data.

Field Name	Data Type	Numeric Range
No.	short int	
rss[i]	double	
Type	short int	1~6
Area	short int	/
Heading	double	60~120 (°)

**Table 8:** Statistical results of turning point recognition.

Trajectory		Turning Points							
		1	2	3	4	5	6	7	8
1	Crossing	3	3	1	2	3	3	5	5
	recognition	3	3	1	2	3	3	5	5
2	Crossing	11	11	3	2	10	6	14	15
	recognition	11	11	3	2	10	6	14	15

**Table 9:** Positioning error statistics with a 2-month interval.

Fingerprint Map	Mean Value [m]	Median [m]	Standard Deviation [m]	80% Percentile [m]
ISFMap	0.87	0.67	0.58	1.24
AUFMap	0.75	0.74	0.46	1.12
CSFMap	0.63	0.50	0.49	0.71

**Table 10:** Positioning error statistics with a 6-mounth interval.

Fingerprint Map	Mean Value [m]	Median [m]	Standard Deviation [m]	80% Percentile [m]
ISFMap	2.87	2.32	2.07	3.67
AUFMap	1.37	1.49	0.77	1.67
CSFMap	0.73	0.66	0.59	1.09

**Table 11:** Positioning error statistics with a 10-mounth interval.

Fingerprint Map	Mean Value [m]	Median [m]	Standard Deviation [m]	80% Percentile [m]
ISFMap	3.57	3.44	3.03	7.32
AUFMap	1.23	1.09	0.70	1.46
CSFMap	0.63	0.50	0.49	0.71

## Data Availability

The original contributions presented in this study are included in the article. Further inquiries can be directed to the corresponding authors.
